# Mapping of CaM, S100A1 and PIP2-Binding Epitopes in the Intracellular N- and C-Termini of TRPM4

**DOI:** 10.3390/ijms21124323

**Published:** 2020-06-17

**Authors:** Kristyna Bousova, Ivan Barvik, Petr Herman, Kateřina Hofbauerová, Lenka Monincova, Pavel Majer, Monika Zouharova, Veronika Vetyskova, Klara Postulkova, Jiri Vondrasek

**Affiliations:** 1 Institute of Organic Chemistry and Biochemistry of the Czech Academy of Sciences, Flemingovo namesti 2, 16000 Prague, Czech Republic; lenka.monincova@uochb.cas.cz (L.M.); pavel.majer@uochb.cas.cz (P.M.); monika.vargova@uochb.cas.cz (M.Z.); veronika.vetyskova@uochb.cas.cz (V.V.); klara.postulkova@uochb.cas.cz (K.P.); jiri.vondrasek@uochb.cas.cz (J.V.); 2 Faculty of Mathematics and Physics, Charles University, Ke Karlovu 5, 12116 Prague, Czech Republic; ibarvik@karlov.mff.cuni.cz (I.B.); herman@karlov.mff.cuni.cz (P.H.); hofbauer@karlov.mff.cuni.cz (K.H.); 3 Institute of Microbiology of the Czech Academy of Sciences, Videnska 1083, 14220 Prague, Czech Republic; 4 Second Faculty of Medicine, Charles University, V Uvalu 84, 150 06 Prague, Czech Republic

**Keywords:** TRPM4 channel, binding epitope, PIP_2_, CaM, S100A1, fluorescence anisotropy, docking, molecular dynamics simulations

## Abstract

Molecular determinants of the binding of various endogenous modulators to transient receptor potential (TRP) channels are crucial for the understanding of necessary cellular pathways, as well as new paths for rational drug designs. The aim of this study was to characterise interactions between the TRP cation channel subfamily melastatin member 4 (TRPM4) and endogenous intracellular modulators—calcium-binding proteins (calmodulin (CaM) and S100A1) and phosphatidylinositol 4, 5-bisphosphate (PIP_2_). We have found binding epitopes at the N- and C-termini of TRPM4 shared by CaM, S100A1 and PIP_2_. The binding affinities of short peptides representing the binding epitopes of N- and C-termini were measured by means of fluorescence anisotropy (FA). The importance of representative basic amino acids and their combinations from both peptides for the binding of endogenous TRPM4 modulators was proved using point alanine-scanning mutagenesis. In silico protein–protein docking of both peptides to CaM and S100A1 and extensive molecular dynamics (MD) simulations enabled the description of key stabilising interactions at the atomic level. Recently solved cryo-Electron Microscopy (EM) structures made it possible to put our findings into the context of the entire TRPM4 channel and to deduce how the binding of these endogenous modulators could allosterically affect the gating of TRPM4. Moreover, both identified binding epitopes seem to be ideally positioned to mediate the involvement of TRPM4 in higher-order hetero-multimeric complexes with important physiological functions.

## 1. Introduction

Transient receptor potential cation channel subfamily melastatin member 4 (TRPM4) is a nonselective monovalent cation channel that is activated and subsequently blocked by intracellular calcium (Ca^2+^) [1] at negative plasma membrane potentials [2,3,4]. TRPM4 contains many regulatory motifs that modulate its Ca^2+^ responsiveness and voltage dependence [1,5,6,7]. Specific mutations in the TRPM4 gene lead to the inhibition of the channel function that directly causes familial cases of heart block disease [5]. Eight structures of the TRPM4 channel have been solved using single-particle cryo-Electron Microscopy (EM) at an overall resolution ranging from 2.9 to 3.8 Å [8,9,10,11]. The TRPM4 structural topology represents a crown-like tetrameric transmembrane core with a domain-swapped architecture [9]. The cytoplasmic N- and C-termini are separated by six transmembrane helices (S1–S6) with a loop between S5 and S6, which works as a selectivity filter [9]. A cluster of hydrophobic amino acids from all four S6 helices forms a lower gate. The channel-gating can be allosterically influenced by endogenous modulators that bind to the intracellular tails of TRPM4. Similarly, the ultimate Ca^2+^ sensitivity is strongly regulated by several intracellular factors, including calmodulin (CaM), phosphatidylinositol 4, 5-bisphosphate (PIP_2_), protein kinase C, ATP, etc. [1,12,13,14].

The cytosolic calcium-binding proteins CaM and S100A1 essentially participate in Ca^2+^ homeostasis in the cell. They are involved in many important cellular pathways due to their interactions with target messengers/receptors. The interactions of CaM or S100A1 with target-binding epitopes have been deeply analysed in the past [15,16,17]. Initially, these interactions are mediated through nonspecific long-range electrostatic interactions of negatively charged residues of CaM or S100A1 and positively charged residues from the binding epitopes of target receptors. Specific contacts are then formed between the so-called hydrophobic anchors from binding epitopes and dedicated hydrophobic cavities of CaM or S100A1. The specific positions of hydrophobic amino acids (either 1-5-10 and/or 1-8-14 [13,17,18,19]) are used for the bioinformatic identification of potential CaM-binding sites [17]. Indeed, several such binding sites have been proposed in the proximal C-terminal region of TRPM4 [12,20,21]. These binding sites seem to be interconnected to each other in a continuous sequence, because a deletion of any region has severely reduced the Ca^2+^ sensitivity of the TRPM4 channel [1].

The PIP_2_ is one of the most abundant intracellular phospholipids [12]; it is also known as an extensive modulator of transient receptor potential channels (TRPs) [22]. Under physiological conditions, PIP_2_ is negatively charged and interacts with positively charged binding epitopes in target proteins. They are often ordered into the so-called Pleckstrin homology (PH) domains with characteristic positions of basic residues [23]. Electrophysiology measurements have proved that the Ca^2+^ sensitivity of desensitised TRPM4 can be recovered by physiological concentrations of PIP_2_ [24]. The proximal TRPM4 N-terminal binding sites for PIP_2_ and PIP_3_ with a modulatory function have recently been confirmed [24,25]. Moreover, the cytosolic C-terminal region proximal to the S1–S4 sensor domain involves a polybasic region that could constitute the PIP_2_-binding site conserved among the TRPC, TRPV and TRPM channels [26]. Based on the cryo-EM structure of TRPM4, it has been proposed that the C-terminal re-entrant segment (protruding into the S1–S4 sensor) of TRPM4 harbours three positively charged arginine residues (R1072, R1086 and R1090) that provide an ideal binding site for membrane-bound PIP_2_ [10]. The binding of PIP_2_ around the S1–S4 sensor domains of different TRPs has been observed in recent cryo-EM structures [27,28,29]. Overall, the region near the S1–S4 sensor domain of TRPM4 appears to be a potential hotspot targeted by competing endogenous modulators (including CaM and PIP_2_) that can affect TRPM4 gating.

Moreover, these endogenous compounds may function as a glue that mediates the incorporation of TRPM4 into hetero-multimeric complexes with unexpected physiological functions. The TRPM4-SUR1-AQP4 complex, involved in brain swelling, has recently been identified [30]. The CaM and PIP_2_ are able to interact with all individual components of the TRPM4-SUR1-AQP4 complex [1,12,31,32,33]. Moreover, the potential binding sites for PIP_2_ and CaM seem to be indiscriminately localised near the cytoplasmic membrane–water interface [34]. Thus, the bi-lobal CaM can potentially bridge and keep together all members of the TRPM4-SUR1-AQP4 complex [35]. In fact, the affinity of TRPM4 to CaM and its sensitivity to intracellular calcium are doubled after TRPM4 co-assembly with SUR1 [31]. Apparently, the TRPM4 antagonists or molecules that somehow disturb the TRPM4-SUR1-AQP4 complex can be used to modulate AQP4 indirectly [30]. Therefore, it is highly needed to decipher the TRPM4-SUR1-AQP4-CaM/PIP_2_ structure at the atomic level.

Here, we have used in vitro and in silico approaches to identify potential CaM-binding epitopes at the intracellular N- and C-termini of TRPM4, which are both proximal to the functionally important S1–S4 sensor domain. Short peptides representing these TRPM4-binding epitopes have been characterised by a fluorescence anisotropy (FA)-binding assay for their ability to anchor various endogenous ligands (CaM, S100A1 and PIP_2_). The importance of representative basic amino acids and their combinations from both TRPM4 peptides for the recognition of the above-mentioned endogenous ligands has been determined by means of site-directed mutagenesis. Computer models of all complexes have been studied using extensive molecular dynamics (MD) simulations. Overall, six shared binding sites for CaM, S100A1 and PIP_2_ at the N- and C-termini of TRPM4 have been identified and characterised. This indicates considerable promiscuity of the potential binding epitopes within the intracellular tails of TRPM4.

## 2. Results

### 2.1. The Design of Peptides Representing Potential TRPM4-Binding Epitopes for CaM, S100A1 and PIP_2_

**Figure 1:**
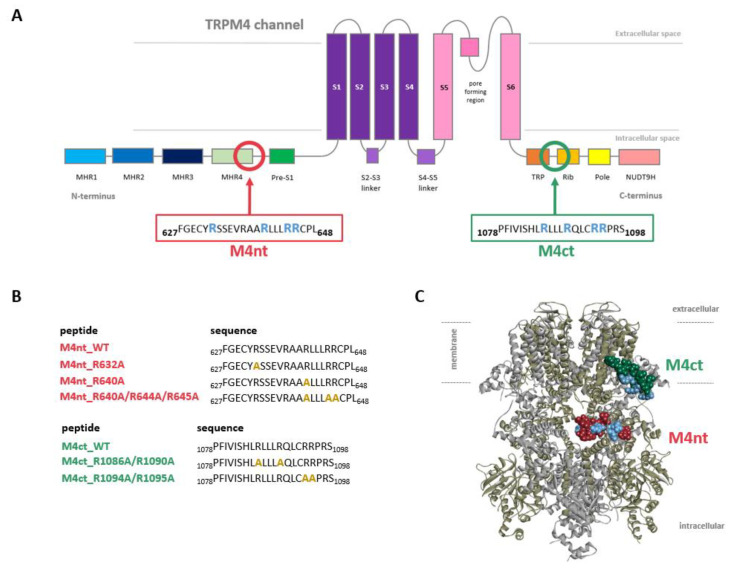
Localisation of transient receptor potential cation channel subfamily melastatin member 4 (TRPM4)-binding epitopes. (**A**) A scheme of the transmembrane topology and domain organisation of the TRPM4. The receptor is composed of six transmembrane helices (S1–S6, violet and pink), with a pore region between the 5th and 6th servers for the transport of monovalent ions (K^+^ and Na^+^). The MHR1–4 and pres-S1 (blue and green) show N-terminal modulatory domains. The Trp, Rib, Pole and NUDT9H (orange, yellow and pink) display the C-terminal modulatory domains. The predicted modulatory binding epitopes—N-terminal (M4nt and F627–L648, deep-pink frame) and C-terminal (M4ct and P1078–S1098, green frame)—display putative calmodulin (CaM), S100A1 and phosphatidylinositol 4, 5-bisphosphate (PIP_2_)-binding epitopes. (**B**) The peptide M4nt and M4ct wild types and their mutant forms synthesised from the designed TRPM4-binding epitopes. The gold alanine residues in the peptide sequences show the mutated positions of the original blue arginine residues. (**C**) The TRPM4 structure side view (6BQV) with M4nt (deep-pink ball representation) and M4ct (green ball representation)-binding epitopes; the blue highlighted (ball representation) arginines display the potential critical basic residues involved in ligand (CaM, S100A1 and PIP2) interactions. The opposite TRPM4 homomeric subunits are shown in the cartoon representation by grey and army colours.

Two potential binding epitopes at the intracellular N- and C- termini of TRPM4 (Figure 1A) were proposed using the Calmodulin Target Database [34]. The N-terminal-binding epitope (positions F627–L648) was represented by the FGECYRSSEVRAARLLLRRCPL peptide (hereinafter termed “M4nt_WT”). The TRPM4 C-terminal-binding epitope (positions P1078–S1098) was represented by the PFIVISHLRLLLRQLCRRPRS peptide (hereinafter termed “M4ct_WT”). The M4nt_WT and M4ct_WT peptides (and their mutants) were used to study their ability to bind CaM, S100A1 and PIP_2_. In vitro FA experiments and in silico molecular modelling (protein–protein docking and MD simulations) were used for this purpose, as described in subsequent paragraphs.

### 2.2. CaM and S100A1 Form Complexes with M4nt_WT and M4ct_WT

**Figure 2:**
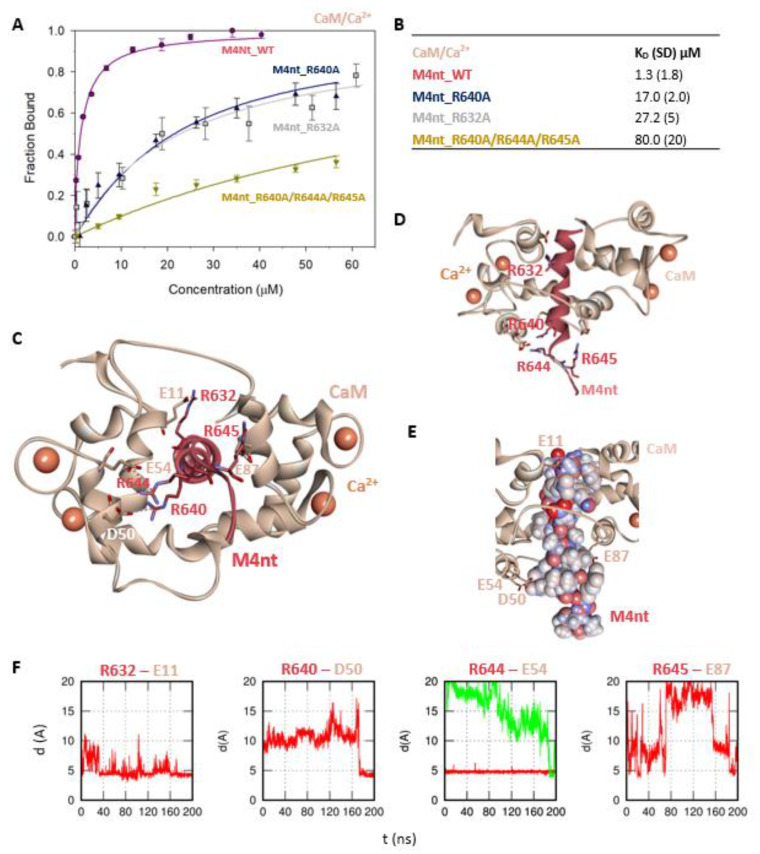
The M4nt/CaM complex. (**A**) The F_B_ of fluorescently labelled M4nt_WT (wild type) (circles), M4nt_R632A (squares), M4nt_R640A (up-triangles) and M4nt_R640A/R644A/R645 (down-triangles) as a function of CaM (beige) concentrations. M4nt peptides were titrated by CaM, and F_B_ was calculated according to Equation (1); the solid lines represent the best fit to the binding isotherm from Equation (2) (Methods). (**B**) The equilibrium K_D_ of the M4nt-binding epitope in complexes with CaM obtained by steady-state fluorescence anisotropy (FA). (**C**) M4nt/CaM in the context of the whole CaM in the presence of Ca^2+^ as a result of molecular dynamics (MDs). The side chains of M4nt (ribbon representation, deep-pink colour) amino acids involved in salt bridges with their binding counterparts from CaM (ribbon representation, beige colour) in atomic detail displayed as sticks: R632-E11, R640-D50, R644-E54 and R645-E87. (**D**) The M4nt/CaM complex from the upper view; M4nt and CaM are shown in the same representation as in Figure 2C, with displayed R632, R640, R644 and R645 basic residues (stick representation, deep-pink colour) involved in the interactions with CaM. (**E**) The M4nt/CaM complex from the upper view; M4nt is shown in CPK representation (partial charge colouring), CaM displayed with E11, D50, E54 and E87 negatively charged residues (stick representation, beige colour) involved in the interactions with M4nt. Ca^2+^ displayed in a ball representation, orange colour. (**F**) Time-dependent geometry characteristics of the studied salt bridges R632-E11, R640-D50, R644-E54 and R645-E87 from MD simulations. A stable salt bridge is expected to have a distance of about 5Å between the termini of oppositely charged amino acids. K_D_: equilibrium dissociation constant.

**Figure 3:**
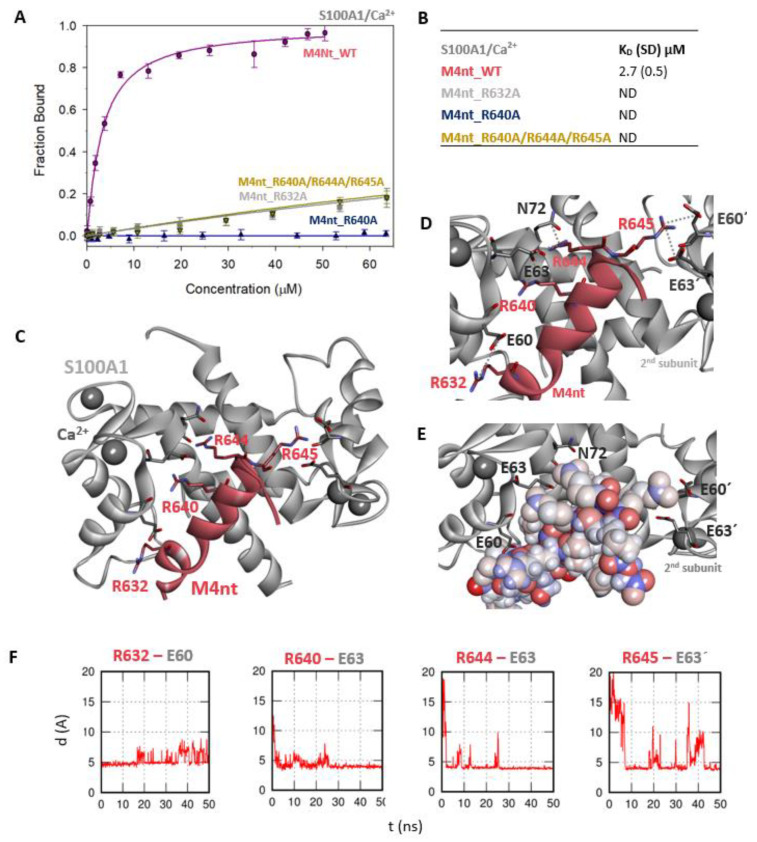
The M4nt/S100A1 complex. (**A**) The F_B_ of fluorescently labelled M4nt_WT (circles), M4nt_R632A (squares), M4nt_R640A (up-triangles) and M4nt_R640A/R644A/R645 (down-triangles) as a function of S100A1 (grey) concentrations. M4nt peptides were titrated by S100A1, and F_B_ was calculated according to Equation 1; the solid lines represent the best fit to the binding isotherm from Equation 2 (Methods). (**B**) The equilibrium K_D_ of the M4nt-binding epitope in complexes with S100A1 obtained by steady-state FA. (**C**) M4nt/S100A1 in the context of the whole S100A1 in the presence of Ca^2+^ as a result of MDs. The side chains of M4nt (ribbon representation, deep-pink colour), with displayed R632, R640, R644 and R645 basic residues involved in the interaction with S100A1 (ribbon representation, grey colour). (**D**) The M4nt/S100A1 complex in detailed view; the binding partners are shown in the same representation as in Figure 3C, with salt bridges in atomic detail displayed as sticks: R632-E60, R640-E63, R644-E63, N72 and R645-E60´ and E63´ of the 2nd monomer. (**E**) The M4nt/S100A1 complex in detailed view; M4nt is shown in CPK (partial charge colouring) representation, S100A1 displayed with E60, E63 and N72 of the 1st monomer and E60´ and E63´ of the 2nd S100A1 monomer with negatively charged residues (stick representation, grey colour) involved in the interactions with M4nt. Ca^2+^ displayed in a ball representation, grey colour. (**F**) Time-dependent geometry characteristics of the studied salt bridges R632-E60, R640-E63, R644-E63 and R645-E63´ from the MD simulations. A stable salt bridge is expected to have a distance of about 5 Å between the termini of oppositely charged amino acids. ND: not determined.

**Figure 4:**
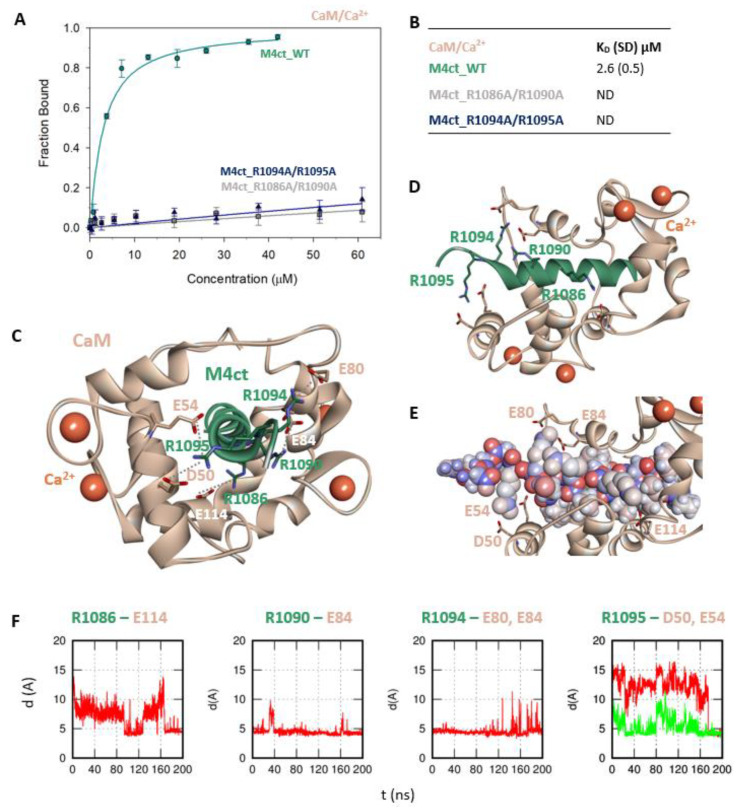
The M4ct/CaM complex. (**A**) The F_B_ of fluorescently labelled M4ct_WT (circles), M4ct_R1086A/R1090A (squares) and M4ct_R1094A/R1095A (up-triangles) as a function of CaM (beige) concentrations. M4ct peptides were titrated by CaM, and F_B_ was calculated according to Equation (1); the solid lines represent the best fit to the binding isotherm from Equation (2) (Methods). (**B**) The equilibrium K_D_ of the M4ct-binding epitope in complexes with CaM obtained by steady-state FA. (**C**) M4ct/CaM in the context of the whole CaM in the presence of Ca^2+^ as a result of MDs. The side chains of the M4ct (ribbon representation, green colour) amino acids involved in salt bridges with their binding counterparts from CaM (ribbon representation, beige colour) in atomic detail displayed as sticks: R1086-E114, R1090-E84, R1094-E80, E84 and R1095-D50 and E54. (**D**) The M4ct/CaM complex from the upper view; M4ct and CaM are shown in the same representation as Figure 4C, with displayed R1086, R1090, R1094 and R1095 basic residues (stick representation, green color) involved in the interactions with CaM. (**E**) The M4ct/CaM complex from a detailed upper view; M4ct is shown in CPK representation (partial charge colouring), CaM displayed with D50, E54, E80, E84 and E114 negatively charged residues (stick representation, beige colour) involved in the interactions with M4ct. Ca^2+^ displayed in a ball representation, orange colour. (**F**) Time-dependent geometry characteristics of the studied salt bridges R1086-E114, R1090-D84, R1094-E80 and E84 and R1095-D50 and E54 from MD simulations. A stable salt bridge is expected to have a distance of about 5Å between the termini of oppositely charged amino acids.

**Figure 5:**
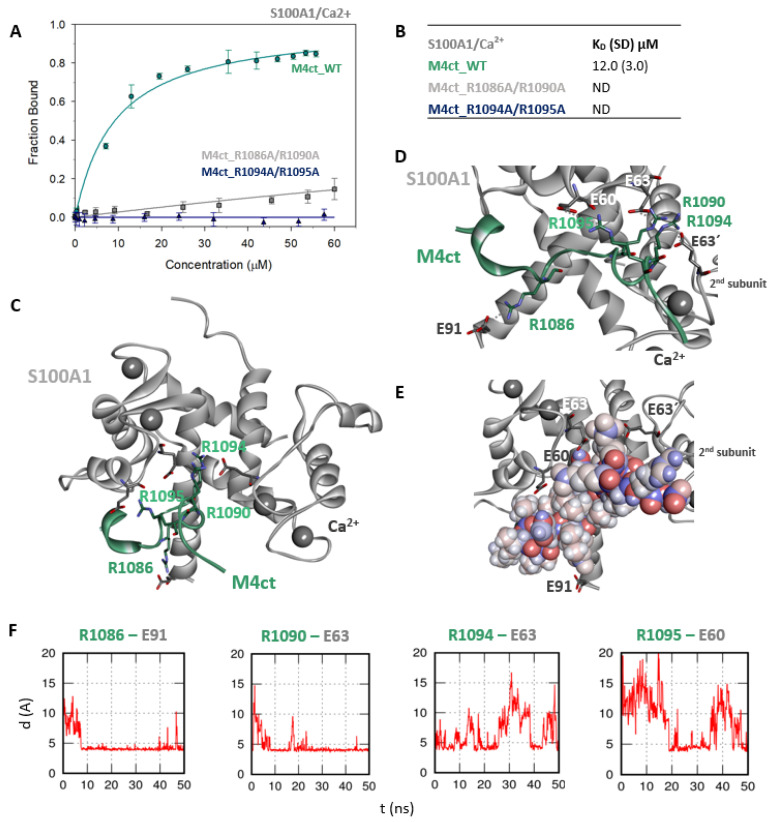
The M4ct/S100A1 complex. (**A**) The F_B_ of fluorescently labelled M4ct_WT (circles), M4ct_R1086A/R1090A (squares) and M4ct_R1094A/R1095A (up-triangles) as a function of S100A1 (grey) concentrations. M4ct peptides were titrated by S100A1, and F_B_ was calculated according to Equation (1); the solid lines represent the best fit to the binding isotherm from Equation (2) (Methods). (**B**) The equilibrium K_D_ of the M4ct-binding epitope in complexes with S100A1 obtained by steady-state FA. (**C**) M4ct/ S100A1 in the context of the whole S100A1 in the presence of Ca^2+^ as a result of MDs. The side chains of M4ct (ribbon representation, green colour) with displayed R1086, R1090, R1094 and R1095 basic residues involved in the interaction with S100A1 (ribbon representation, grey colour). (**D**) The M4ct/S100A1 complex in detailed view; the binding partners are shown in the same representation as in Figure 5C, with salt bridges in atomic detail displayed as sticks: R1086-E91, R1090-E63, R1094-E63 and R1095-E60 and R1090- and R1094-E63´ of the 2nd monomer. (**E**) The M4ct/S100A1 complex in detailed view; M4ct is shown in CPK (partial charge colouring) representation, S100A1 displayed with E60, E63 and N72 of the 1st monomer and E63´ of the 2nd S100A1 monomer, with negatively charged residues (sticks representation, grey colour) involved in the interactions with M4ct. Ca^2+^ displayed in a ball representation, grey colour. (**F**) Time-dependent geometry characteristics of the studied salt bridges R1086-E91, R1090-E63, R1094-E63 and R1095-E60 from MD simulations. A stable salt bridge is expected to have a distance of about 5Å between the termini of oppositely charged amino acids.

First, we studied whether M4nt_WT and M4ct_WT are able to bind CaM and S100A1 at all. The carboxyfluorescein-labelled M4nt_WT and M4ct_WT peptides were titrated with increasing amounts of CaM or S100A1, and FA was measured for each point of titration. The FA increased due to a slower rotational diffusion of the formed complexes, and the fraction bound (F_b_) of M4nt_WT and M4ct_WT could be calculated (Figure 2A, Figure 3A, Figure 4A, Figure 5A). Since the specific interactions of M4nt_WT and M4ct_WT with CaM/S100A1 were calcium-dependent [36,37,38,39,40], all samples were measured in the presence of 200-μM CaCl_2_. The fluorescence lifetimes τ_i_ of all peptides and their complexes (and all the following characterised peptides/complexes) were constant during all the experiments, with the values close to 3 ns. All complexes were characterised using their dissociation constants (K_D_): M4nt_WT/CaM with K_D_ = 1.3 (SD 1.8) μM, M4nt_WT/S100A1 with K_D_ = 2.7 (SD 0.5) μM, M4ct_WT/CaM with K_D_ = 2.6 (SD 0.5) μM and M4ct_WT/S100A1 with K_D_ = 12 (SD 3.0) μM (Figure 2B, Figure 3B, Figure 4B, Figure 5B). Overall, the FA results indicated the high affinity of M4nt_WT and M4ct_WT peptides to CaM and S100A1. Both M4nt_WT and M4ct_WT peptides thus seemed to represent potential binding epitopes on TRPM4.

### 2.3. The Basic Amino Acids of M4nt_WT and M4ct_WT are Crucial for Binding to CaM and S100A1

Further, we examined the involvement of selected basic residues in interactions (presumably salt bridges) stabilising complexes of both peptides with CaM and S100A1. We were interested in the positional dependence of these interactions and their potential cooperativity. Therefore, several representative analogues of both peptides by alanine scanning mutagenesis were prepared (see Figure 1B: M4nt_R632A, M4nt_R640A, M4nt_R640A/R644A/R645A, M4ct_R1086A/R1090A and M4ct_R1094A/R1095A). For M4nt_R632, an approx. 20-fold decrease of binding affinity to CaM with K_D_ = 27.2 (SD 5) µM (Figure 2B) was observed. For M4nt_R640A, there was an approx. 13-fold decrease of binding affinity to CaM with K_D_ = 17 (SD 2) µM. The triple-mutant M4nt_R640A/R644A/R645A confirmed that basic amino acids work cooperatively, because about 62-fold higher K_D_ = 80 (SD 20) µM than in the case of M4nt_WT was determined. Moreover, the M4nt_R632A, M4nt_R640A and M4nt_R640A/R644A/R645A mutant peptides were not able to bind S100A1 at all (Figure 3B). Further, a total loss of M4ct_R1086A/R1090A and M4ct_R1094A/R1095A-binding to CaM and S100A1 were observed with K_D_ ≫ 250 µM—i.e., in the “not determined” (ND) range (Figure 4B and Figure 5B). The maximum concentrations of the proteins and peptides were, in some cases, limited by precipitation and their solubility; therefore, it was not possible to achieve the saturation (plateau), basically. To summarise, the FA measurements confirmed that all the studied basic amino acids of both peptides substantially stabilised their complexes with CaM and S100A1 (Figure 2A,B, Figure 3A,B, Figure 4A,B, Figure 5A,B).

### 2.4. The Binding Interfaces of M4nt_WT/CaM and M4ct_WT/CaM Complexes

The binding interfaces of M4nt_WT/CaM and M4ct_WT/CaM were studied in detail by in silico molecular modelling (i.e., by protein–protein docking and MD simulations). Initially, both peptides (preorganised into α-helices) were docked into various CaM structures that had originally been complexed with peptides carrying hydrophobic anchors in the canonical positions 1–10 (3SUI) [41], 1–14 (1CDL) [42] and 1–17 (2BCX) [43]. The amino acid sequences of M4nt_WT and M4ct_WT peptides contain many bulky hydrophobic amino acids that can serve as hydrophobic anchors orienting peptides properly with respect to CaM. Nevertheless, the phenylalanines at the N-termini of both peptides (i.e., F627 in M4nt_WT and F1079 in M4ct_WT) are best suited for this purpose. Therefore, antiparallel complexes where these N-terminal phenylalanines were buried into the hydrophobic cavity in the C-domain of CaM were selected as the most appropriate for the positioning. Subsequently, the M4nt_WT/CaM and M4ct_WT/CaM complexes were relaxed by means of extensive MD simulations.

More specifically, the protein–protein docking of M4nt_WT into the 1–17 (2BCX) structure of CaM provided two complexes that corresponded to the canonical hydrophobic binding motifs 1–14/1–17 and 1–10. The R632, R644 and R645 of M4nt_WT were involved in the salt bridges formed with E11, D50, E54 and E87 of CaM (according to conventional numbering). During the MD simulations, all the studied basic amino acids of M4nt_WT (i.e., R632, R640, R644 and R645) were significantly involved in the salt bridges with the acidic residues E11, D50, E54 and E87 of CaM (Figure 2C–F, Figure S1).

The protein–protein docking of M4ct_WT into the 1–14 (1CDL) structure of CaM resulted in a complex with the hydrophobic-binding motif 1–7/1–10. A similar complex was obtained by the docking of M4ct_WT into the 1–17 (2BCX) structure of CaM. However, only R1090 and R1094 of M4ct_WT formed salt bridges with their acidic counterparts of CaM. Nevertheless, within subsequent MD simulations, all the studied basic amino acids (i.e., R1086, R1090, R1094 and R1095) successfully found their acidic counterparts D50, E54, E80, E84 and E114 in CaM (Figure 4C–F, Figure S2).

To summarise, M4nt_WT/CaM and M4ct_WT/CaM complexes have been obtained. All the studied basic amino acids formed salt bridges with CaM (regardless of their initial conformation) during MD simulations. This indicates their importance, explored by alanine mutants, which lost their affinity, demonstrated by apparent increases in the K_D_ values.

### 2.5. The Binding Interfaces of M4nt_WT/S100A1 and M4ct_WT/S100A1 Complexes

In addition, both M4nt_WT and M4ct_WT peptides were docked into the S100A1 structures (2KBM and 2K2F) [44], which had originally been complexed with peptides representing the TRTK12 and RyRP12-binding epitopes.

The most reasonable complex identified by the ClusPro protein–protein docking process revealed that the M4nt_WT peptide was bound to the main binding site of S100A1 [40,43]. During subsequent MD simulations, M4nt_WT retained the canonical alpha-helical conformation, and numerous stabilising salt bridges were established (Figure 3C–F). The M4nt_WT R632 formed a salt bridge with E60 from the first monomer of S100A1. The same applied for the R640 and R644 of M4nt_WT, forming salt bridges with E63 from the first monomer of S100A1. Moreover, the R644 interaction with N72 from the first monomer of S100A1 was observed. There were two additional salt bridges between R645 and E60’ and E63’ of the second monomer of S100A1. This means that M4nt_WT was able to bridge both S100A1 subunits. Furthermore, the K56 of S100A1 from the first monomer was bound to the acidic E635 of M4nt_WT.

The M4ct_WT/S100A1 complex identified by the ClusPro web server was completely consistent with known crystal structures in the sense that the hydrophobic L1087 of M4ct_WT was directed to the binding site defined by the V57, L77 and L81 side chains of S100A1. It was similar to RyRP12 peptide binding in the 2K2F structure [44]. However, the number of stabilising salt bridges substantially increased within a subsequent MD run (Figure 5C–F). In fact, all the studied basic amino acids were found to be bound by salt bridges to the first monomer of S100A1: R1086-E91, R1090-E63, R1094-E63 and R1095-E60. Moreover, R1090 and R1094 also formed salt bridges with E63’ of the second monomer of S100A1.

Analogously to the previous binding of M4nt and M4ct to CaM, M4nt_WT/S100A1 and M4ct_WT/S100A1 complexes were obtained that led to the establishment of numerous salt bridges being stable during the simulation. In both cases studied, the importance of basic amino acids in the binding epitope was proved.

### 2.6. M4nt_WT and M4ct_WT Bind PIP_2_

**Figure 6:**
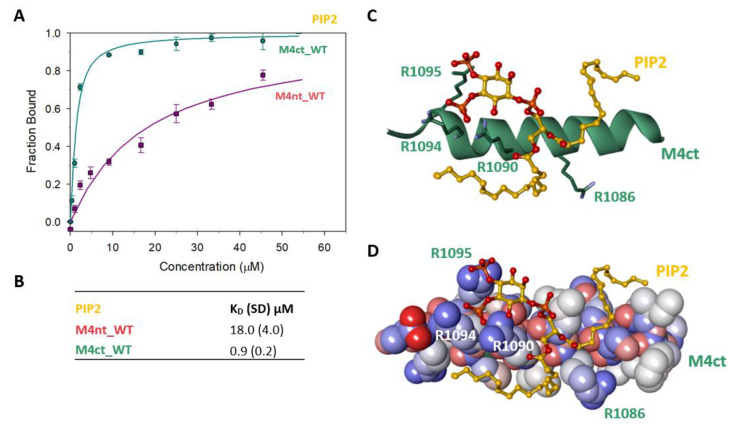
The M4nt/PIP_2_ and M4ct/PIP2 complexes. (**A**) The F_B_ of M4ct_WT (circles) and M4nt_WT (squares) as a function of PIP_2_ (yellow) concentrations. The labelled PIP_2_ was titrated by M4nt or M4ct peptides, and F_B_ was calculated according to Equation (1); the solid lines represent the best fit to the binding isotherm from Equation (2) (Methods). (**B**) The equilibrium K_D_ of PIP_2_ in complexes with M4nt and M4ct peptides obtained by steady-state FA. (**C**,**D**) Spontaneous association of PIP_2_ (ball and stick representation, yellow colour) and M4ct (sticks in green colour or CPK with a partial-charge representation) was observed within a 100-ns MD run. The salt bridges were formed between the phosphate groups of PIP_2_ and R1090, R1094 and R1095 of M4ct (green colour, stick representation).

To identify the M4nt_WT/PIP_2_ and M4ct_WT/PIP_2_ complexes, we again used steady-state FA measurements with PIP2, labelled as TopFluor^®^ PI (4, 5) P2 (Avanti polar lipids, Alabaster, Alabama 35007-9105, USA). The PIP_2_ was titrated with increasing amounts of nonlabelled M4nt_WT or M4ct_WT, and FA was measured for each point of titration. The fraction bound (F_b_) was calculated for M4nt_WT and M4ct_WT (Figure 6A). The complexes were characterised by the determination of K_D_. The K_D_ of the M4nt_WT/PIP_2_ complex was 18.0 (SD 4.0), whereas the K_D_ of the M4ct_WT/ PIP_2_ complex was 0.9 (SD 0.2) μM (Figure 6B).

### 2.7. The Binding Interface of the PIP_2_/M4ct_WT Complex

To determine the positioning of the PIP2 in the M4ct_WT/PIP_2_ complex, ten independent MD runs were performed, each lasting 100 ns. The total length of the MD trajectories reached 1 µs. There was no interaction between the M4ct peptide and the PIP2 detected at the beginning of the MD simulations. M4ct_WT and PIP2 were separated by bulk water molecules. The first contacts between PIP_2_ and M4ct_WT were established relatively quickly, usually after about 10 ns, and electrostatic interactions were substantially involved in this process. PIP_2_ had three phosphate groups with a total charge of −5. M4ct_WT carried the exact opposite charge, +5. Additional stabilisation can originate from contacts of hydrophobic lipid tails of PIP_2_ with hydrophobic amino acids of M4ct_WT. A typical result of the MD simulations is depicted in Figure 6C,D. In this particular case, salt bridges were formed between phosphate groups of PIP_2_ and arginine side chains of M4ct_WT (R1090, R1094 and R1095—involved in CaM/S100A1 complexes).

## 3. Discussion

The modulation of receptors by CaM and S100A1 is well-described in the scientific literature [20,39,43]. For the RyR1 receptor, two overlapping binding epitopes have been characterised for CaM and S100A1 [45]. The competition between CaM and S100A1 for the overlapping binding epitopes of TRPs and other receptors (e.g., RyR1) has already been investigated [39,44,45,46,47]. In vitro experiments have previously confirmed the competition of CaM and S100A1 at the very same concentration levels [46].

In the presented study, we have decided to investigate whether CaM, S100A1 and PIP_2_ can also share binding epitopes in TRPM4. The Ca^2+^-dependent formation of protein/peptide complexes has been described many times [39,47]. The in vitro Ca^2+^ concentrations used to stimulate the M4/CaM/S100A1 interactions were hence maintained at the same concentration level of 200 µM. Two potential binding epitopes for the endogenous modulators of TRPM4 have been identified in the N- and C-termini of TRPM4-representing peptides. The binding affinities of the M4nt_WT and M4ct_WT peptides to endogenous modulators were examined in vitro, which revealed a typical micromolar range of K_D_ values [36,40,46,47,48]. The N-terminal binding site (M4nt_WT) is apparently able to bind CaM and S100A1; moreover, the C-terminal binding site (M4ct_WT) binds PIP_2_ as well. The relative occupancy of endogenous modulators will depend on their actual concentration in the intracellular environment. Regarding the lower physiological concentrations of S100A1 as compared to the abundant CaM [49], we suppose that, in vivo, both potential binding epitopes in TRPM4 will be occupied predominantly by CaM.

Generally, the binding of CaM and S100A1 to epitopes involves two distinct driving forces. The first is mostly of long-range electrostatic and rather nonspecific character, resulting in salt bridges with the involvement of long and flexible arginine side chains acting in synergy. The second driving force is apparently of Van der Waals origin, in which the most important role is played by dispersion. Ultimately, the so-called hydrophobic anchors of peptides are properly placed into the binding cavities of CaM and S100A1. Here, we have only addressed the importance of electrostatic forces by means of FA measurements and arginine-to-alanine mutagenesis. A set of representative basic amino acids and their combinations was used. Nevertheless, computer modelling was based on the profound bioinformatics analysis of both peptides to identify all potential hydrophobic anchors situated in well-known canonical positions. Based on that, we usually chose as reliable only those results of protein–protein docking where bulky hydrophobic anchors of M4nt_WT/M4ct_WT peptides were buried into the binding cavities of CaM/S100A1. Overall, our docking results showed that both M4ct_WT and M4nt_WT peptides bind to CaM in an antiparallel manner. Finally, we allowed all complexes to relax by means of extensive MD simulations. Thanks to this, we have obtained final models where all mutated basic amino acids of both peptides have found their acidic binding partners in CaM/S100A1/PIP2 endogenous modulators of TRPM4.

The currently known structures of TRPM4 obtained at the resolution of ∼2.88−3.8 Å [8,9,11] have suggested that the so-called TRP domain (i.e., W1058–R1098, which includes M4ctWT-P1078–1098) bridges the gating helix S6 and cytoplasmic C-terminal Rib helix. This domain is considered as a key determinant for signal transduction and channel gating [10]. The TRP domain is divided into two characteristic segments, the first of which is the helical stretch running parallel to the cytosolic surface of the membrane as an extension of S6, labelled as the TRP helix. The second TRP domain segment is a re-entrant loop and helix. The re-entrant loop (P1073–P1077) is embedded between helices S1 and S2 of the transmembrane S1–S4 sensor domain, whereas the re-entrant helix (P1078–R1098, i.e., M4ct) is located at the cytoplasmic side. The M4ct segment is thus perfectly positioned to interact with such membrane-embedded molecules as PIP_2_. It has been also described that the TRPC3 C-terminal loop (connecting the re-entrant and Rib helix) affects channel gating by altering the allosteric coupling between the cytoplasmic and transmembrane domains [50]. Electrophysiological analyses have disclosed that the shortening of the length of the C-terminal loop increases TRPC3 activity and that the elongation of the length of the loop has the opposite effect. The C-terminal moiety of TRPM4 was proposed to be a target of CaM already in several previous studies [1,20,21]. However, all cryo-EM structures of TRPM4 show the re-entrant helix embedded in the membrane—i.e., not readily available for CaM. Nevertheless, the moiety is a common feature of TRPM, TRPC and TRPV channels. In several TRPC structures, this segment has not been resolved, which indicates conformational disorder [51]. More interestingly, a comparison of available TRPV2 structures (see the amino acids 675–684 in 5HI9 [51] and 6BWM [52]) shows that, under suitable conditions, the re-entrant moiety can be re-localised into the cytoplasm, and it is available for modulatory bindings. This probably allows interactions with endogenous modulators other than the membrane-anchored PIP_2_ (including CaM and S100A1). The M4nt_WT is in proximal contact with the so-called Rib helix, which forms another binding site for CaM, confirmed by experiments in TRPM4 [1], as well as in structurally homologous TRPC channels [53]. An examination of the currently available TRPM4 structures has led us to the conclusion that the M4nt_WT and M4ct_WT segments are close enough to simultaneously bind one CaM molecule. This means that M4nt_WT might serve as a base for conformational changes stimulated by the CaM of the re-entrant moiety, which includes M4ct_WT.

The C-terminal segment of TRPM4 was previously designed as a target for PIP_2_ [10]. Our results provide a detailed description of the binding interface. The structure of TRPV5-PIP_2_ (PDB: 6DMU) discloses the binding site between the N-linker, S4-S5linker and S6-helix of TRPV5 [28]. These interactions with PIP_2_ induce conformational rearrangements in the lower gate, resulting in channel activation. Furthermore, based on the TRPM4-SUR1-AQP4 complex, we assume that PIP_2_ could potentially keep M4ct_WT and the TMD0 of SUR1 domains together, as seen in the Kir6.2-SUR1 complex [54]. Interestingly, the M4ct_WT-PIP_2_ interactions could be physiologically relevant in a specialised cardiac conduction system and Purkinje fibres [55]. Mutations in the TRPM4 gene (including mutations in the C-terminal part of TRPM4) have been reported to cause familial cases of progressive cardiac conduction disease and heart block [5]. In particular, I1082S and R1086G mutants were identified when a total of 330 cases of sudden, unexpected deaths were tested for cardiac channelopathy and cardiomyopathy genes [55].

## 4. Conclusions

Since TRPM4 has been designated as a functional agent of various diseases, including cancer and heart attack [3,5], it is necessary to describe in detail its function and modulation mechanisms. In this work, we describe two new binding sites at the N- and C-termini of the channel. In particular, the M4nt_WT and M4ct_WT intracellular segments of TRPM4 have been confirmed as shared binding epitopes for commonly known [26,46] endogenous modulators such as CaM, S100A1 and PIP_2_. The shared, so-called promiscuous characters of some ligand-binding sites is a known feature of many proteins [56,57,58,59]. The receptor-binding segment exploits its disorder character to be more flexible and adaptable to bind different types of ligands. Each ligand with a diverse binding affinity to the receptor domain induces distinct structural changes in the channel, resulting in a different functional response and channel modulation. The novel TRPM4 N- and C-terminal promiscuous binding sites for CaM, S100A1 and PIP2 are promising candidates for the diverse modulation of the channel. Such ligands are commonly utilised by cells as activators and/or inhibitors of the functions of many receptors. The effect and strength of the regulation depends on the character of an acceptor (receptor) binding interface and the structural changes induced within. CaM, S100A1 and PIP2 have been proven as effective modulatory molecules of many receptors [20,26,39]. Moreover, the effect of the modulation can be multiplied across shared ligand-binding sites via more channel subunits [15], indicating a very complex regulatory process that occurs during receptor communication with the external environment. We suppose that these new M4nt/CaM-, M4nt/S100A1-, M4nt/PIP2-, M4ct/CaM-, M4ct/S100A1- and M4ct/PIP2-binding interfaces described in atomic detail will help to clarify multicomplex TRPM4 modulatory functions and will stimulate further functional studies of the whole TRPM4 by in vivo assays with the listed promising ligands.

## 5. Materials and Methods

### 5.1. Design of TRPM4 N- and C- Terminal Binding Epitopes

For the identification of novel CaM-binding motifs commonly defined by the hydrophobic positions 1-5-10 and 1-10-14 or by the IQ motif at the intracellular N- and C- termini of human TRPM4 (UniProtKB/Swiss-Prot: Q8TD43), we used the Calmodulin Target Database [34]. This tool was also utilised to identify a S100A1-binding epitope because it is known that this ligand recognises the binding motif at the receptor, very often overlapping with a CaM-binding site with the same or very similar hydrophobic positions [40,45]. Furthermore, we have also identified the potential PIP2-binding sites using PH-domain characteristics [60,61].

### 5.2. M4nt and M4ct Peptide Synthesis and Site-Directed Mutagenesis

M4nt and M4ct peptides and their alanine-scanning analogues were synthesised by solid-phase peptide synthesis according to the Nα-Fmoc protocol in our previous publication [40].

### 5.3. CaM, S100A1 Purification and PIP2 Preparation

CaM and S100A1 cDNAs were subcloned into the pET28b expression vector, and they were expressed and purified according to our standard purification protocol [40]. The fluorescently labelled PIP2, 1-oleoyl-2- {6- [4- (dipyrrometheneboron difluoride) butanoyl] amino}x hexanoyl-sn-glycero-3-phosphoinositol-4,5-bisphosphate, shortly TopFluor^®^ PI(4,5)P2, was purchased from Avanti Polar Lipids, Inc. (Alabaster, AL, USA).

### 5.4. Steady-State Fluorescence Anisotropy

The experiments were performed on a K2 spectrofluorometer (ISS, Inc., Champaign, IL, USA) at 25 °C in a cuvette with a 2-mm path length. The FA-binding assays were performed in a 25-mM Tris-HCl (pH 7.5) buffer containing 250-mM NaCl and 200-µM CaCl_2_. M4nt and M4ct peptides were labelled by carboxyfluorescein and titrated by small aliquots of the ligands (CaM and S100A1). In the opposite way, labelled PIP_2_ was titrated with nonlabelled M4 peptides. Fluorescence was excited at 490 nm, and polarised emission components *I*_II_ and *I*_⊥_, required for the construction of the emission anisotropy, were acquired and averaged quasi-simultaneously at 525 nm by repetitive switching of the emission polariser. Any residual scattered light was suppressed by a long-pass dielectric filter (520 nm) placed in front of the input slit of the emission monochromator. The anisotropy values *r* were calculated from the fluorescence intensities in the parallel (*I*_II_) and perpendicular (*I*_⊥_) directions according to the relationship *r* = (*I*_II_ − *GI*_⊥_)/(*I*_II_ + 2*GI*_⊥_) [62], where *G* is a factor correcting for different transmittance of the detection channel for the two measured polarisations (*I*_II_ and *I*_⊥_). The G factor was determined in a separate experiment. The measurements were repeated six times for each ligand concentration; the mean anisotropy value was calculated and used for further analysis. The fractions of bound ligands F_B_ were evaluated as [63]:

(1) $$F_{B}=(r\mathrm{obs}\;-\;r\min)\;/\;[(r\max\;-\;r\mathrm{obs})Q+(r\mathrm{obs}\;-\;r\min)],$$

where Q is the quantum-yield ratio of the bound to the free form of the labelled peptide; *r*_max_ is the anisotropy of the complex at saturation; *r*_min_ is the minimum anisotropy for free M4nt, M4ct or PIP_2_ and *r*_obs_ is the measured anisotropy at any intermediate ligand concentration. The Q was evaluated for every binding experiment from the ratio of the fluorescence lifetimes of the bound to the free M4nt, M4ct or PIP_2_: Q = *τ*_bound_/*τ*_free_. For the determination of the equilibrium dissociation constant (K_D_), F_B_ was plotted as a function of the ligand concentration and fitted by [64]:

(2) $$F_{B}\;=\frac{K_{D}\;+\left[P1\right]+\left[P2\right]\;-\sqrt{{\left(K_{D}\;+\left[P1\right]+\left[P2\right]\right)}^{2}-4\left[P1\right]\left[P2\right]}}{2\left[P1\right]},$$

where [*P*1] represents the M4nt, M4ct or PIP_2_ concentration, and [*P*2] is the ligand concentration. Nonlinear data fitting was performed using SigmaPlot 11.0 (Systat software, Inc., San Jose, CA 95110, USA).

### 5.5. Time-Resolved Fluorescence Measurements

Fluorescence lifetimes were evaluated at room temperature in a drop placed on a coverslip and inserted in an inverted confocal microscope IX83 (Olympus, Hamburg, Germany) equipped with TimeHarp 260 PICO time-correlated single-photon counting electronics and cooled GaAsP hybrid detectors (all PicoQuant, Berlin, Germany). The M4 peptides or PIP_2_ fluorescence were excited at 485 nm by an LDH-485 picosecond laser head (PicoQuant, Berlin, Germany). Emission decays were collected in the epifluorescence mode using a combination of a 488-nm dichroic reflector (Olympus, Hamburg, Germany) and a Semrock 520/35 bandpass filter in the detection path. Fluorescence was assumed to decay multiexponentially according to the formula:

(3) I(t)=∑iαi×exp(−tτi)

where *τ_i_* are fluorescence lifetimes and *α_i_* the corresponding amplitudes. The intensity-weighted mean fluorescence lifetime was calculated as:

(4) $$\tau_{mean}=\underset{i}{\sum }\alpha_{i}\times \tau_{i}^{2}/\;\underset{i}{\sum }\alpha_{i}\times \tau_{i}$$

The least-squares deconvolution fitting was performed by the SymPho Time 64 software (PicoQuant).

### 5.6. Protein–Protein Docking

The ClusPro web server was used for the docking of α-helical M4nt_WT/M4ct_WT peptides (created using the Molefacture module in VMD studio) [65] into the CaM and S100A1 structures. The ClusPro 2.0: protein-protein docking server was chosen based on the results of the community-wide contest called CAPRI (Critical Assessment of Predicted Interactions), in which ClusPro is traditionally doing well [66]. The quality of the very fast, fully automated and reproducible docking produced by ClusPro is very close to that of the best human predictor groups, which can use any type of information [67].

The ClusPro server performs rigid-body docking by sampling billions of conformations by means of the PIPER docking program [68], which is based on the Fourier transform (FFT) correlation approach. RMSD-based clustering of the 1000 lowest-energy structures generated makes it possible to find the centres of the largest clusters that represent the most likely models of the complex. Selected structures are refined using energy minimisation.

### 5.7. Molecular Dynamics Simulations

The protein–peptide complexes obtained by protein–protein docking were simulated by means of extensive MD sampling at different temperatures. The MD simulations of the selected complexes utilised the AMBER_ILDN (The Amber Project, San Francisko CA 94158-2517, USA) force field [69], and water molecules were modelled using the Transferable Intermolecular Potential 3-Point (TIP3P) water model [70]. Prior to the production of MD simulations, all systems were energy-equilibrated using the *pmemd* module of AMBER 14 [71]. MD runs (lasting for 50–1800 ns) were performed with the *pmemd.cuda.MPI* module of AMBER 14, which runs exclusively on GPUs at the equivalent speed of tens of standard processor cores [72]. The SPFP precision model was used, and periodic boundary conditions (PBC) were applied. The particle-mesh Ewald (PME) method was used for the calculation of electrostatic interactions [73]. A cut-off distance of 8 Å was applied for Lennard-Jones interactions. The temperature was maintained at 300K via Langevin dynamics with a friction factor of 5. The Monte Carlo barostat (a new addition to AMBER 14), which samples rigorously from the isobaric–isothermal ensemble, was used for the production phase. The covalent bonds involving hydrogen atoms were constrained using the SHAKE algorithm. For water molecules, a special “three-point” RATTLE algorithm was used [74]. The hydrogen mass repartitioning scheme allowed a timestep set to 4 fs [75]. Data were recorded every 100 ps.

The PIP_2_-M4ct_WT complex was explored by long MD runs. The simulated complex was solvated using a (TIP3P) water model [70] to ensure at least 10 Å of solvent in the periodic box and neutralised in 0.5-M NaCl. This gave a periodic box with a size of ~ 60 × 60 × 60 Å for a simulated system consisting of ~19,000 atoms. All-atom structure and topology files were generated using VMD [63]. Forces were computed using a CHARMM36 forcefield for proteins, lipids and ions [76]. MD simulations were produced by means of the software package NAMD2.13 [77], running on workstations equipped with NVIDIA graphics processing units. Simulated systems were energy-minimised and heated to 300 K. Langevin dynamics were used for temperature control, with the target temperature set to 300 K, and the Langevin piston method was applied to reach an efficient pressure control with a target pressure of 1 atm [77]. The production of the MD runs was 100 ns. The integration timestep was set to 2 fs. The SETTLE algorithm (tolerance, 1 × 10^−8^) was applied to constrain the bonds in the water molecules [78]. The nonbonded cut-off was set to 11 Å. Data were recorded every 20 ps. All MD trajectories were visualised with the aid of the VMD 1.9 software package [65] and analysed by means of the CPPTRAJ module from the AMBER Tools suite [79]. The figures were produced using the Biovia Discovery Studio [80].

## Supplementary Materials

Supplementary materials can be found at https://www.mdpi.com/1422-0067/21/12/4323/s1. Time evolution of salt bridges in M4nt and M4nt interactions with CaM indicating formation of salt bridges between termini of oppositely charged amino acids both binding partners (Figures S1 and S2).

## Abbreviations

ATP	Adenosine triphosphate
CaM	Calmodulin
EM	Electron microscopy
FA	Fluorescence anisotropy
FFT	Fourier transform
MD	Molecular dynamics
PBC	Periodic boundary conditions
PH	Pleckstrin homology
PIP_2_	phosphatidylinositol 4, 5-bisphosphate
PME	particle mesh Ewald
S100A1	S100 calcium-binding protein A1
TRPs	transient receptor potential (channels)
TRPC	TRP canonical
TRPV	TRP vaniloid
TRPM4	TRP melastatin 4
