# Supplementary material for: Mapping of CaM, S100A1 and PIP2-Binding Epitopes in the Intracellular N- and C-Termini of TRPM4
Source: Int J Mol Sci. 2020 Jun 17;21(12):4323. doi: 10.3390/ijms21124323 (PMC7352223; doi:10.3390/ijms21124323)
Supplement: Supplementary file 1 [file ijms-21-04323-s001.pdf]

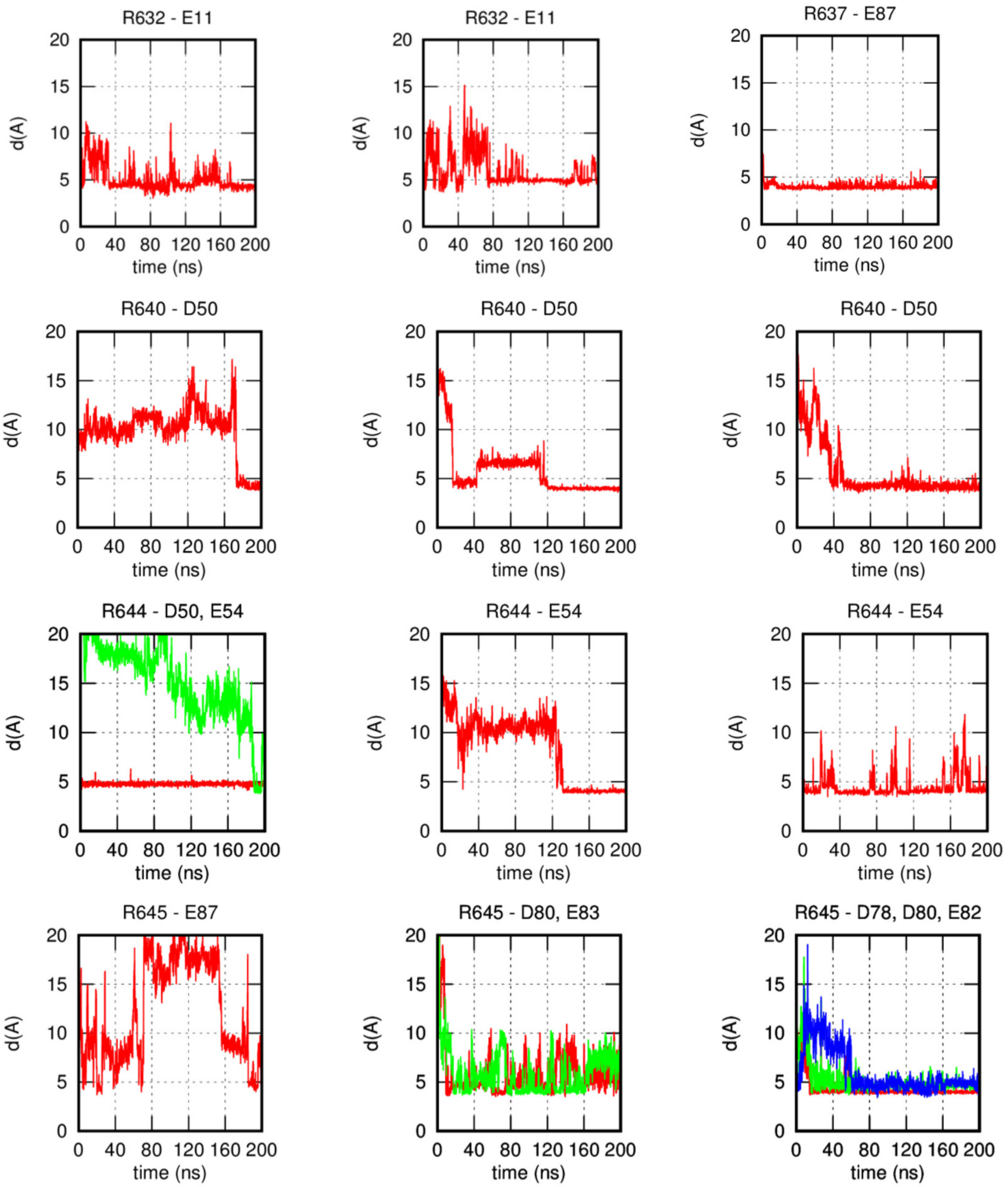

FIGURE S1 Time evolution of salt bridges in M4nt/CaM within three independent MD runs. Distances about 5A indicate formation of salt bridges between termini of oppositely charged amino acids**.**

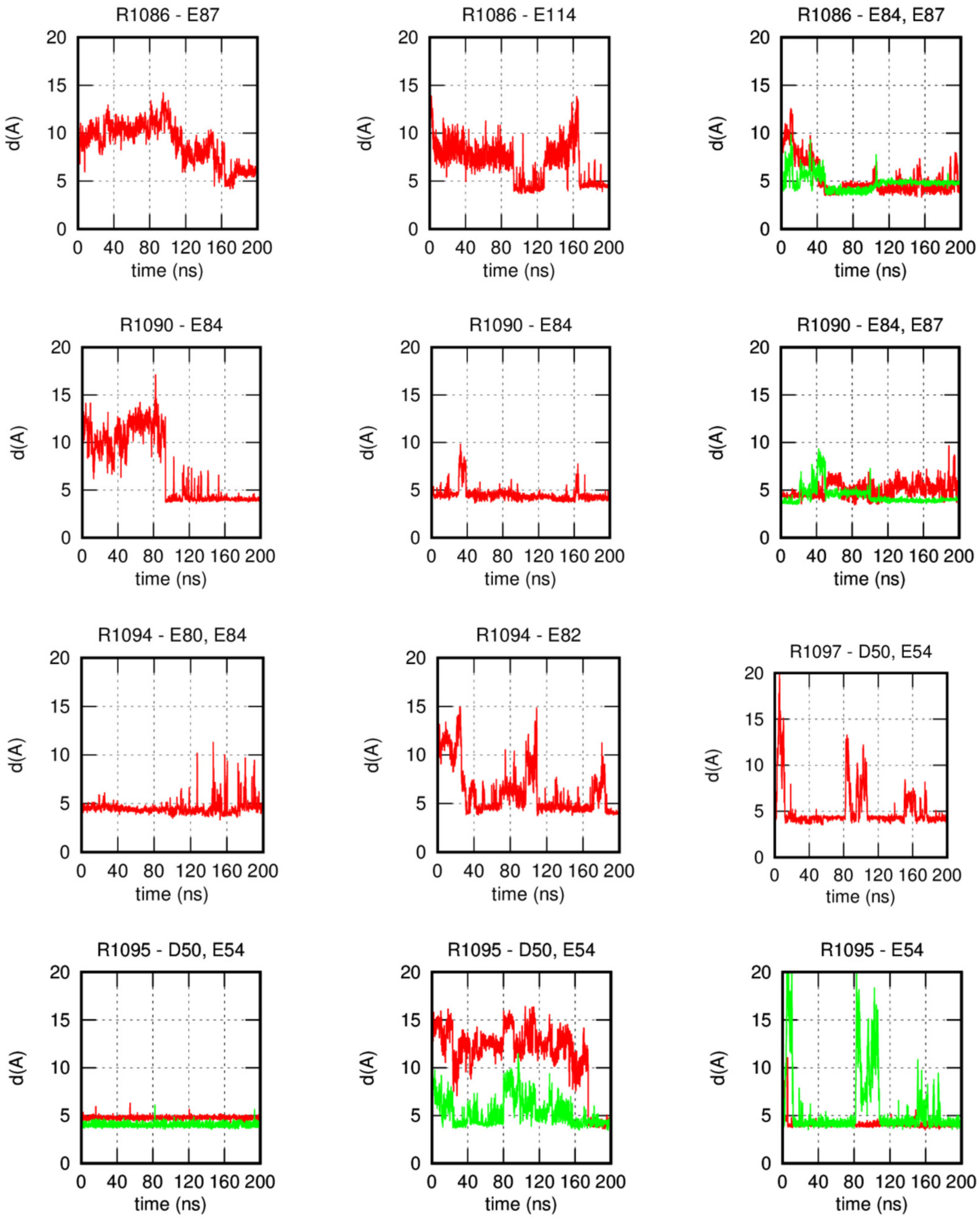

FIGURE S2 Time evolution of salt bridges in M4ct/CaM within three independent MD runs. Distances about 5A indicate formation of salt bridges between termini of oppositely charged amino acids.
